# EFFECTIVE CLINICIAN BEHAVIORS FOR INCLUDING FAMILY CAREGIVERS IN THE GERIATRIC MENTAL HEALTH SETTING

**DOI:** 10.1093/geroni/igad104.1884

**Published:** 2023-12-21

**Authors:** Mary Wyman, Hayley Raj, Joseph Perzynski, Carey Gleason, Ranak Trivedi, Amy Byers, Corrine Voils

**Affiliations:** Madison VA/Univ Wisconsin, Madison, Wisconsin, United States; University of Wisconsin, Madison, Wisconsin, United States; Madison VA Hospital, Madison, Wisconsin, United States; UW Madison, Madison, Wisconsin, United States; Stanford University, Stanford, California, United States; UCSF / San Francisco VA Health Care System, San Francisco, California, United States; W.S. Middleton Memorial Veterans Hospital, Madison, Wisconsin, United States

## Abstract

To improve patient and caregiver outcomes, the 2022 National Strategy to Support Family Caregivers recommends training for professionals on caregiver inclusion and provision of person- and family-centered care. Caregiver inclusion is especially important for older adults with dementia, who experience high rates of comorbid mental health (MH) disorders but are disadvantaged by disparities in care access and quality. Current training options are limited. Our VA-funded project seeks to develop caregiver inclusion training for MH clinicians working with older adults with dementia. We present preliminary findings from in-depth, 45-min qualitative interviews with 9 multidisciplinary MH clinicians (psychiatry, social work, psychology, and clinical pharmacy) on their experiences with engaging family caregivers in MH treatment. A multidisciplinary coding team conducted content analysis to identify facilitators and barriers to effective communication and collaboration with caregivers, with a focus on clinician behaviors. Facilitators of effective caregiver involvement include clinician attention to developing rapport with the caregiver, addressing knowledge gaps about dementia and mental health interventions, and helping to address the challenges of caregiving with supportive resources. Barriers included not tailoring clinician approach to caregiver needs, failing to clarify the caregiver role in healthcare, and failing to address one own’s defensiveness or labeling of caregivers in distress. The importance of system factors (e.g., technology, clinic scheduling, and care team dynamics) was noted. Findings will be used to inform the development of strategies to increase clinician skills for caregiver inclusion, supporting improved patient and caregiver outcomes and the goals of the RAISE Act and the National Strategy.

